# 

**Published:** 1916-06-10

**Authors:** 


### CONTENTS.

PAGE

**Remedies for the Neglect of Science** ... ... 225

**The Penal Powers of the General Medical Council** ... ... ... ... ... ... 226

**Hospital and Institutional News** ... ... ... 227
The New Chelsea Hospital for Women—The Birthday Honours—The Teaching of Medical Ethics—Wastage of Army Doctors—A Carnt Memorial at Manchester—Hospitals and Those Who Make Them—A King's Fund for Provincial Centres—Hospital Zeppelin Finance—Naval Ambulance Train on View—Yorkshire and the Arc-en-Barrois Hospital—The Star and Garter Home—The Proposed Radium Institute for Birmingham—Death of the Founder of the American Hospital in France—A Lack of Patriotism at the Belgrave Hospital—Hospital Staffs Before the Tribunals—The Taxpayers *versus* the Discharged Soldier—Hospital Work at the Cape—Calcutta Hospital for Tropical Diseases—Infantile Mortality—Lupus in School Children—Vicarious Economy—An Opportunity for Doncaster—Tuberculosis Activities in the Midlands—The Prevention of Measles—The Incineration of Household Refuse—The Flight of a Patient—The *Southern Cross* in June—An Epsom Hostel—This Week's Drug Market.

**On Ocular Symptoms in Tabes Dorsalis** ... 233

Local Symptoms of a General Import.

**Fire Extinguishers: A Point for Institutional Managers** ... ... ... ... ... ... 234

**Human Temperaments** ... ... ... ... 235

IV.—Cleverness and Capability.

**Hæmaturia in Tumours of the Bladder** ... 236

**The College of Nursing Meeting** ... ... ... 237
Matters to be Discussed.
Qualifications for Admission to the Register.

PAGE

**The King's Birthday Honours** ... ... ... 238
Naval and Military Lists.

**The School Doctor** ... ... ... ... ... ... 239

The School Doctor and the Mentally Defective Child (*continued*).

**Hospital Architecture and Construction** ... 240
Hospital for Tropical Diseases, Calcutta.

**Buildings Contemplated** ... ... ... ... 240

**Munificent Gift to Cardiff Hospital** ... ... 240

**The Matrons' and Sisters' Department** ... 241
The College of Nursing: Some Absurdities and a Warning.
The Spirit of Compromise.
Round the Hospitals.
The Dangers of Haste—The Registration Fee—What Constitutes a Training School?—Factors in the Training of Nurses—The Birthday Honours.

**The Editor's Letter-Box** ... ... ... ... 243
The English of Medical Writings—Economy in the Annual Report.

**The Book Market** ... ... ... ... ... 243

**Parliament and the Profession** ... ... ... 244

**The London Hospital** ... ... ... ... 245

Points from the Quarterly Report.

**Passing Events and Topics** ... ... ... ... 245

**Hospital Results in 1915** ... ... ... ... 246

**Auxiliary and other Military Hospitals** ... 246

Sandon Hall, Stafford.

**Matrons' and Sisters' Appointments** ... ... vi

**Gifts to Hospitals** ... ... ... ... ... vi

**The Institutional Worker** ... ... (Suppl.) 1
The Nursing Organisation of a Children's Open-air Ward.

